# Changes in fetal and neonatal mortality during 40 years by offspring sex: a national registry-based study in Norway

**DOI:** 10.1186/1471-2393-13-101

**Published:** 2013-05-02

**Authors:** Fredrik Carlsen, Jostein Grytten, Anne Eskild

**Affiliations:** 1 Department of Economics, Norwegian University of Science and Technology, Trondheim, Norway; 2 Department of Community Dentistry, University of Oslo, Oslo, Norway; 3 Department of Obstetrics and Gynecology, Institute of Clinical Medicine, Akershus University Hospital, Lørenskog, Norway; 4 Department of Mental Health, Norwegian Institute of Public Health, Oslo, Norway

**Keywords:** Fetal mortality, Neonatal mortality, Convergence in mortality

## Abstract

**Background:**

There has been a considerable decline in fetal and neonatal mortality in the Western world. The authors hypothesized that this decline has been largest for boys, since boys have a higher risk of fetal and neonatal death.

**Methods:**

The authors used data from the Medical Birth Registry about all births in Norway to study changes during 1967–2005 in mortality for boys and girls from the 23rd week of pregnancy until one month after birth. Absolute and relative yearly changes in fetal and neonatal death rates were estimated separately for boys and girls.

**Results:**

From 1967 to 2005, the average annual reduction in the overall death rate was greater for boys: 0.47 per 1000 boys (95% CI: 0.45, 0.48) and 0.37 per 1000 girls (95% CI: 0.35, 0.39). These estimates were not affected by adjustments made for changes over time in maternal characteristics. The convergence in death rates by sex was strongest for the first week after birth: average annual reduction in the early neonatal death rate was 0.24 per 1000 boys (95% CI: 0.23, 0.25) and 0.17 per 1000 girls (95% CI: 0.16, 0.18). The death rates for boys and girls also converged during pregnancy and from one week to one month after birth. The relative reduction in death rates was quite similar for boys and girls: the overall death rate fell annually by 4.4% (95% CI: 4.3, 4.6%) for boys and by 4.2% (95% CI: 4.0, 4.4%) for girls.

**Conclusions:**

During the period 1967–2005, the absolute reduction in fetal and neonatal death rates was greatest for boys. The relative reduction in mortality was about the same for both sexes, but the absolute reduction was greatest for boys since the mortality for boys began at a higher level. The convergence of death rates was not due to changes in the composition of mothers, suggesting that convergence has been caused by technological progress.

## Background

During the last decades, there has been a considerable decline in fetal and neonatal mortality in the Western world [1-4]. Improvements in antenatal, obstetric and infant care are likely to be the major causes [5-8]. In Norway during the last forty years, the fetal death rate has declined by 70 per cent in pregnancies lasting longer than 22 weeks [9]. There has also been a large decline in neonatal mortality [10]. Boys have an increased risk of fetal and neonatal death and are over-represented among offspring with an increased risk of fetal and neonatal death such as preterm delivery or growth restriction [11,12]. Since the risk of fetal and neonatal death for boys is higher than for girls, we hypothesized that the secular decline in fetal and neonatal mortality has been largest for boys.

Among all deliveries by Norwegian women during the period 1967–2005, we studied whether the decline in fetal and neonatal death rates differed between boys and girls. Separate analyses were performed for pre-term births, term births, the early neonatal period and the late neonatal period.

## Methods

### Design

We performed a population-based follow-up study by utilizing data from two national Norwegian registers: the Medical Birth Registry of Norway [13] and the Norwegian Central Person Registry [14].

### Study population

Since 1967, all births after 16 weeks of gestation have been reported to the Medical Birth Registry of Norway. Our study population comprised all births from 23 weeks of gestation during the period 1967 to 2005, a total of 2,263,736 births. We excluded all mothers that immigrated to Norway (N = 144,858). We want to focus on trends in long term mortality caused by improvements in maternity care and infant care. From 1967 to 2005, the proportion of deliveries by immigrant mothers increased from 2.3% to 15.6%. Children of immigrant mothers have, on average, higher mortality than the rest of the population, and there has been considerable variation over time in the countries that immigrants to Norway come from [15,16]. Inclusion of births by immigrant mothers would therefore make the sample more heterogeneous and complicate interpretation of results. Information on the mother’s country of origin was obtained by linking the Medical Birth Registry with the Norwegian Central Person Registry using the mother’s unique identification number.

Pregnancies recorded as lasting more than 43 weeks were excluded (N = 27,341), since gestational length may have been miscoded in some of these pregnancies. We could not with certainty determine which were miscoded, so we excluded all these pregnancies. Also, we omitted births with unknown sex of the child (N = 260) and without information on gestational age at birth (N = 116,466) leaving 1,974,811 births for the analysis.

### Study factors

Gestational age at birth was calculated from the date of the last menstrual period for births during the years 1967 to 1998. For the years 1999 to 2005, gestational age at birth was based on estimates of term date at routine fetal ultrasonographical examinations in weeks 17–19 of pregnancy. Information from ultrasonographical examinations was available for 97.6% of the women, and for the remaining women the date of the last menstrual period was used. Information on vital status at birth was obtained from the Medical Birth Registry. Information about infant death and the age of the infant at death were obtained from the Central Person Registry.

We adjusted for maternal age, parity, plurality and maternal education at delivery, since these factors are associated with offspring death [17,18] and the composition of mothers with regard to these factors has changed during our study period. Information on maternal age (coded: five-year intervals), parity (coded: first child, other children) and plurality (coded: single, multiple births) was obtained from the Medical Birth Registry. Information about maternal education (coded: compulsory school only, upper secondary education, university/college education, unknown education) was obtained from the Education Registry [19]. All educational institutions in Norway report yearly individual data to the Education Registry. Hence, we were able to obtain individual information about the highest education of the mother at the time of the delivery.

### Statistical methods

We carried out separate analyses for each of the following periods of pregnancy: 23–28 weeks, 29–36 weeks and 37–43 weeks. In each analysis, only fetuses that were alive in utero at the beginning of the period were included. The outcome variable distinguished between pregnancies where the fetus died in utero or during birth and pregnancies where the offspring was born alive or was still alive in utero by the end of each period of pregnancy. For infants who were born alive, we did separate analyses for the following time periods after birth: ≤ 1 week and 1 week-1 month. For these analyses the outcome variable distinguished between infants who died during the period and infants who were alive at the end of the period.

To estimate absolute yearly change in fetal and neonatal death rates, the following linear probability model [20] was fitted for each of the three pregnancy periods and two neonatal periods, and for the whole period from the 23rd week of pregnancy to one month after birth:

$$\begin{array}{l} {\mathrm{Mortality}}_{\mathrm{it}}=\alpha_{1}{\mathrm{Boy}}_{\mathrm{it}}+\alpha_{2}{\mathrm{Girl}}_{\mathrm{it}}\\ \;+\alpha_{3}{\mathrm{Boy}}_{\mathrm{it}}\left({\mathrm{Year}}_{t}-1967\right)\\ \;+\alpha_{4}{\mathrm{Girl}}_{\mathrm{it}}\left({\mathrm{Year}}_{t}-1967\right)\\ \;+\alpha_{5}{\mathrm{Boy}}_{\mathrm{it}}{\left({\mathrm{Year}}_{t}-1967\right)}^{2}\\ \;+\alpha_{6}{\mathrm{Girl}}_{\mathrm{it}}{\left({\mathrm{Year}}_{t}-1967\right)}^{2}+\epsilon_{\mathrm{it}}, \end{array}$$

where Mortality_it_ is a dummy variable that is one if fetus/infant *i* in year *t* died during the period and zero otherwise, Boy_it_ (Girl_it_) is a dummy variable turned on if the fetus/infant was a boy (girl), ϵ_it_ is an error term, and α_1_ – α_6_ are parameters to be estimated. α_1_ can be interpreted as the predicted death rate for boys in 1967, that is, the death rate that would have prevailed if the error term had been zero. α_2_ is the corresponding predicted death rate for girls in 1967. The predicted death rates in year t are α_1_ + (t-1967) α_3_ + (t-1967)^2^ α_5_ for boys and α_2_ + (t-1967) α_4_ + (t-1967)^2^ α_6_ for girls, implying that predicted death rates fell by 38 α_3_ + 38^2^ α_5_ for boys and 38 α_4_ + 38^2^ α _6_ for girls during 1967–2005. The absolute yearly change in death rates in year t is α_3_ + 2 (t-1967) α_5_ for boys and α_4_ + 2 (t-1967) α_6_ for girls. α_3_ and α_4_ can be interpreted as absolute yearly changes in death rates in 1967. In 2005, absolute yearly changes in death rates were α_3_ + 76 α_5_ for boys and α_4_ + 76 α_6_ for girls.

We also estimated a simplified linear probability model where α_5_ and α_6_ are set equal to zero. In the simplified specification, absolute yearly changes in death rates are constant and equal to α_3_ for boys and α_4_ for girls. The estimates of α_3_ and α_4_ in this model can therefore be interpreted as average yearly changes in absolute death rates during the whole period from 1967 to 2005.

Since there is considerable random variation between years in death rates, we report predicted rather than actual death rates for individual years. Adjustment for the effects of maternal age, parity, plurality and maternal education on absolute yearly changes in death rates was made by including these variables as covariates in the linear probability models.

To obtain estimates of relative yearly change in death rates, the following logistic regression model was fitted:

$$\begin{array}{l} \mathrm{Prob}\left({\mathrm{Mortality}}_{\mathrm{it}}=1\right)=\Lambda \left[\alpha_{1}+\alpha_{2}{\mathrm{Boy}}_{\mathrm{it}}\right)\\ \;+\alpha_{3}{\mathrm{Boy}}_{\mathrm{it}}\left({\mathrm{Year}}_{t}-1967\right)\\ \;\left(+\alpha_{4}{\mathrm{Girl}}_{\mathrm{it}}\left({\mathrm{Year}}_{t}-1967\right)\right], \end{array}$$

where Λ is the logistic cumulative distribution function [20]. Exp(α_2_)-1 can be interpreted as predicted excess death rate for boys in 1967, whereas exp(α_3_)-1 and exp(α_4_)-1 are the relative yearly change in death rates for boys and girls respectively. Adjusted estimates of relative yearly changes were obtained by including maternal factors as covariates in the logistic regression model.

We estimated the sex-specific number of fetal and infant deaths prevented from 1967 to 2005 by comparing two scenarios: one scenario where, for each year from 1967–2005, mortality was set equal to predicted mortality for 1967, and another scenario where mortality was set equal to predicted mortality in the actual year. In these calculations, the simplified linear probability model was used.

The study was approved by the Regional Committee for Medical and Health Research Ethics (Reference number 603-07276a 1.2007.2366).

## Results

During the study period 1967 to 2005, fetal mortality was higher for boys than for girls in pre-term pregnancies (Table 1). For term pregnancies, boys and girls had similar mortality rates. Neonatal mortality was considerably higher for boys than for girls.

**Table 1:** Fetal and neonatal deaths and death rates by sex of child for non-immigrants, 1967-2005

	**Up to birth**						**After birth**			
	**23 - 28 weeks**		**29-36 weeks**		**≥ 37 weeks**		**≤ 1 week**		**1 week - 1 month**	
	**Boys**	**Girls**	**Boys**	**Girls**	**Boys**	**Girls**	**Boys**	**Girls**	**Boys**	**Girls**
Number at risk	1,015,083	959,728	1,014,279	959,035	1,012,028	957,188	1,009,708	954,987	1,004,798	951,578
Number of deaths	804	693	2,251	1,847	2,320	2,201	4,910	3,409	959	637
Percentage deaths	0.08	0.07	0.22	0.19	0.23	0.23	0.49	0.36	0.10	0.07

### Overall decline in mortality

**Figure 1:**
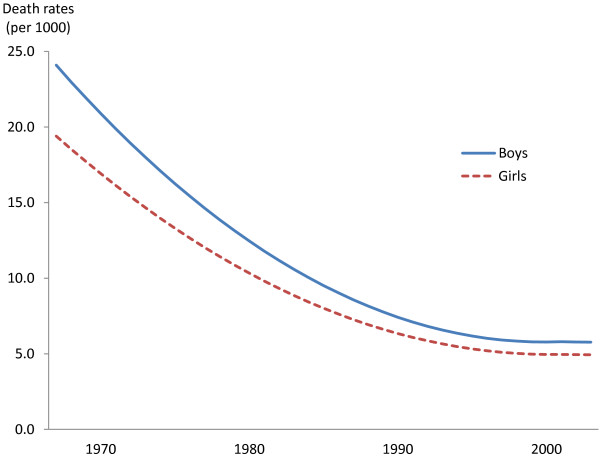
Estimated time trends of overall death rates for boys and girls.

Table 2 shows results for the overall death rate from the 23rd week of pregnancy to one month after birth. The first column presents results for the linear probability model where mortality is quadratic in time. Figure 1 presents the corresponding time trends in death rates. In 1967, the overall death rate was higher for boys than for girls; 24.1 predicted deaths per 1000 boys (95% CI: 23.6, 24.6), 19.4 predicted deaths per 1000 girls (95% CI: 18.9, 19.9). The yearly reduction in absolute death rates in 1967 was 1.12 per 1000 boys (95% CI: 1.05, 1.18), 0.87 per 1000 girls (95% CI: 0.80, 0.94). By 2005, the fall in overall mortality had stopped as yearly changes in absolute death rates were close to zero.

**Table 2:** Absolute change (per 1000) and relative change in mortality from pregnancy week 23 to 1 month after birth by sex of child for non-immigrants in Norway, 1967–2005

	**Absolute change**				**Relative change**	
	**Coefficient**	**95% CI**	**Coefficient**	**95% CI**	**Coefficient**	**95% CI**
Boy	24.094	23.567, 24.622	19.647	19.272, 20.002	1.245	1.191, 1.301
Girl	19.406	18.863, 19.949	15.958	15.583, 16.334	1.000	
Boy * (Year - 1967)	−1.116	−1.183, -1.048	−0.466	−0.483,−0.449	0.956	0.954, 0.957
Girl * (Year-1967)	−0.867	−0.936, −0.798	−0.370	−0.387, −0.353	0.958	0.956, 0.960
Boy * (Year - 1967)^2^	0.017	0.015, 0.019				
Girl * (Year - 1967)^2^	0.013	0.011, 0.015				
Test for equal average trends for boys and girls:			p < 0.001		p = 0.132	
N				1,974,811		

Linear probability and logistic regression analyses. Pregnancies with gestational length >22.

The second column in Table 2 shows results for the simplified linear probability model where mortality is linear in time. During the years 1967–2005, average annual reduction in the overall death rate was greater for boys than for girls; 0.47 per 1000 boys (95% CI: 0.45, 0.48), 0.37 per 1000 girls (95% CI: 0.35, 0.39). Compared to the linear probability model where mortality is quadratic in time, the simplified model predicts lower absolute death rates in 1967 and 2005. The two models produce almost identical estimates of changes in death rates between 1967 and 2005. The parameters of the full linear probability model implies that the death rate for boys fell by 1.79 percentage points from 1967 to 2005, whereas the death rate for girls fell by 1.42 percentage points in the same period. The corresponding estimates from the simplified model are 1.77 and 1.41 percentage points. The two models also produce similar results for the fetal and neonatal periods we consider. To conserve space, we therefore present results for the simplified model only in the rest of the Results section.

The annual relative change in the death rate was about the same for boys and girls (the third column of Table 2). The death rate for boys fell annually by 4.4% between 1967 and 2005 (95% CI: 4.3, 4.6%), and the death rate for girls fell by 4.2% annually (95% CI: 4.0, 4.4%).

When the maternal factors, age, parity, plurality and education at delivery, were included as covariates, the estimated annual reductions in absolute and relative death rates changed very little (Table 3).

**Table 3:** Adjusted absolute change (per 1000) and adjusted relative change in mortality from pregnancy week 23 to 1 month after birth by sex of child for non-immigrants in Norway, 1967–2005

	**Absolute change**		**Relative change**	
	**Coefficient**	**95% CI**	**Coefficient**	**95% CI**
Boy	19.647	19.052, 20.242	1.242	1.188, 1.298
Girl	15.958	15.356, 16.560	1.000	
Boy * (Year - 1967)	−0.460	−0.477, −0.442	0.958	0.956, 0.960
Girl * (Year - 1967)	−0.365	−0.383, −0.357	0.960	0.958, 0.962
Test for equal average trends for boys and girls:	p < 0.001		p = 0.132	
N	1,974,811			

Linear probability and logistic regression analyses. Adjustment made for maternal age (five-year intervals), parity (first child, other children), plurality (single, multiple births) and maternal education (compulsory school only, upper secondary education, university/college education, unknown education).

### Decline in fetal mortality

In 1967, boys had the highest fetal mortality. The predicted death rates in 1967 were (all per thousand): 1.1 for boys (95% CI: 1.0, 1.2) and 1.0 for girls (95% CI: 0.9, 1.1) in weeks 23–28, 3.8 for boys (95% CI: 3.7, 4.0) and 3.4 for girls (95% CI: 3.3, 3.6) in weeks 29–36, and 4.1 for boys (95% CI: 3.9, 4.3) and 3.9 for girls (95% CI: 3.8, 4.1) in weeks 37–43 (Table 4).

**Table 4:** Absolute change (per 1000) and relative change in fetal mortality rates by sex of child for different pregnancy periods

	**23-28 weeks**				**29-36 weeks**				**≥ 37 weeks**			
	**Absolute change**		**Relative change**		**Absolute change**		**Relative change**		**Absolute change**		**Relative change**	
	**Coefficient**	**95% CI**	**Coefficient**	**95% CI**	**Coefficient**	**95% CI**	**Coefficient**	**95% CI**	**Coefficient**	**95% CI**	**Coefficient**	**95% CI**
Boy	1.146	1.046, 1.247	1.184	0.997, 1.407	3.847	3.680, 4.013	1.102	1.000, 1.214	4.105	3.930, 4.280	1.062	0.969, 1.164
Girl	0.982	0.879, 1.086	1.000		3.441	3.270, 3.612	1.000		3.943	3.763, 4.123	1.000	
Boy * (Year - 1967)	−0.019	−0.024, −0.015	0.975	0.969, 0.981	−0.089	−0.096, −0.081	0.959	0.955, 0.962	−0.099	−0.107, −0.091	0.955	0.951, 0.959
Girl * (Year - 1967)	−0.014	−0.019, −0.009	0.980	0.974, 0.987	−0.082	−0.090, −0.075	0.955	0.951, 0.959	−0.089	−0.098, −0.081	0.960	0.956, 0.963
Test for equal average trends for boys and girls:	p = 0.131		p = 0.142		p = 0.274		p = 0.242		p = 0.119		p = 0.081	
N	1,974,811				1,973,314				1,969,216			

Non-immigrants in Norway, 1967–2005. Linear probability and logistic regression analyses. Pregnancies with gestational length >22 weeks.

The annual reduction in death rates during 1967–2005 was 0.019 per 1000 boys (95% CI: 0.015, 0.024) and 0.014 per 1000 girls (95% CI: 0.009, 0.019) in weeks 23–28, 0.089 per 1000 boys (95% CI: 0.081, 0.096) and 0.082 per 1000 girls (95% CI: 0.075, 0.090) in weeks 29–36 and 0.099 per 1000 boys (95% CI: 0.091, 0.107) and 0.089 per 1000 girls (95% CI: 0.081, 0.098) in weeks 37–43 (Table 4).

### Decline in early neonatal mortality

In 1967, the predicted death rates during the first week after birth were 9.3 of 1000 new-born boys (95% CI: 9.1, 9.5) and 6.7 of 1000 new-born girls (95% CI: 6.5, 6.9) (Table 5). From 1967 to 2005, the yearly reduction in the death rate was greater for boys than for girls; 0.24 per 1000 boys (95% CI: 2.3, 2.5) and 0.17 per 1000 girls (95%: CI: 1.6, 1.8). The relative yearly reduction in the death rate was 5.3% for boys (95% CI: 5.0-5.6%), and 5.1% for girls (95% CI: 4.8-5.4%) (Table 5).

**Table 5:** Absolute change (per 1000) and relative change in neonatal mortality by sex of child for non-immigrants in Norway, 1967–2005

	**≤ 1 week**				**1 week - 1 month**			
	**Absolute change**		**Relative change**		**Absolute change**		**Relative change**	
	**Coefficient**	**95% CI**	**Coefficient**	**95% CI**	**Coefficient**	**95% CI**	**Coefficient**	**95% CI**
Boy	9.305	9.067, 9.542	1.406	1.314, 1.504	1.360	1.255, 1.465	1.393	1.176, 1.649
Girl	6.705	6.460, 6.949	1.000		0.972	0.864, 1.080	1.000	
Boy * (Year - 1967)	−0.241	−0.252, −0.230	0.947	0.944, 0.950	−0.022	−0.027, −0.017	0.977	0.971, 0.982
Girl * (Year - 1967)	−0.170	−0.182, −0.159	0.949	0.946, 0.952	−0.016	−0.021, −0.011	0.975	0.969, 0.982
Test for equal average trends for boys and girls:	p < 0.001		p = 0.253		p = 0.115		p = 0.729	
N	1,964,695				1,956,376			

Linear probability and logistic regression analyses. Pregnancies with gestational length > 22 weeks.

### Decline in late neonatal mortality

In 1967, the predicted late neonatal death rate was 1.4 per 1000 boys (95% CI: 1.3, 1.5), and 1.0 per 1000 girls (95% CI: 0.9, 1.1) (Table 5). The annual reduction in the death rate was 0.022 per 1000 boys (95% CI: 0.017, 0.027) and 0.016 per 1000 girls (95% CI: 0.017, 0.027). The relative yearly reduction in the death rate was 2.3% for boys (95% CI: 1.8, 2.9%), and 2.5% for girls (95% CI: 1.8, 3.1%) (Table 5).

### Prevented numbers of fetal and neonatal deaths

Based on the results for absolute changes in death rates (Tables 4 and 5), we estimate that since 1967, a total of 4,109 fetal deaths among boys have been prevented (386 in pregnancy weeks 23–28, 1,767 in pregnancy weeks 29–36 and 1,956 in pregnancy weeks 37–43) (Table 6). A total of 3,504 fetal deaths have been prevented among girls (269 in pregnancy weeks 23–28, 1,556 in pregnancy weeks 29–36 and 1,679 in pregnancy weeks 37–43) (Table 6). The corresponding figures for neonatal deaths were 5,192 prevented deaths among boys (4,767 early neonatal deaths and 425 late neonatal deaths) and 3,489 prevented deaths among girls (3,187 early neonatal deaths and 302 late neonatal deaths) (Table 6).

**Table 6:** Reduction in stillbirths and neonatal deaths 1967–2005 for non-immigrant population due to downward trends in fetal and neontal mortality

	**Up to birth**			**After birth**		**Total**
	**23-28 weeks**	**29-36 weeks**	**≥ 37 weeks**	**≤ 1 week**	**1 week - 1 month**	
Boys	386	1,767	1,956	4,767	425	9,301
Girls	269	1,556	1,679	3,187	302	6,993
Difference	117	211	277	1,580	123	2,308

Based on estimates reported in Tables 4 and 5 (absolute changes).

Hence, the total number of prevented deaths was 9,301 for boys and 6,993 for girls, giving a sex difference in prevented deaths of 2,308. Of all prevented deaths, 68% of the sex difference (1,580/2,308) could be explained by the greater reduction in early neonatal mortality for boys, 26% (605/2,308) could be explained by the greater reduction in fetal mortality for boys and 5% (123/2,308) by the greater reduction in late neonatal mortality for boys.

## Discussion

During the period 1967–2005, the fetal and neonatal death rates in Norway fell more for boys than for girls. Thus, since 1967 the difference in mortality by sex has been reduced, and higher number of deaths among boys than girls has been prevented. The relative reduction in death rates during the period was, however, about the same for boys and girls.

### Strengths/limitations

The strength of this study is the population design with information about mortality for all births in Norway during almost 40 years. We had information up until 2005. The lack of data for recent years is due to the time-consuming process of updating national registries.

We lack data on the length of gestation at birth for about five per cent of pregnancies. Both mortality and the proportion of boys are somewhat higher for these pregnancies than for the pregnancies included in our sample. The exclusion of pregnancies of unknown length could have biased the estimates of trends in excess fetal mortality for boys. However, we were able to study how exclusion of these pregnancies influenced trends in excess neonatal mortality for boys. We repeated the analysis of neonatal mortality with the extra pregnancies included, and the results turned out to be very similar to the results presented in Table 5. Hence, for neonatal mortality, exclusion of pregnancies of unknown length did not bias our results.

### Other studies

We are not aware of other research in which sex-specific trends in fetal mortality and neonatal mortality have been estimated and compared.

### Interpretation

During the last decades, there has been a large decline in fetal and neonatal mortality [1-4]. There has also been an increase in maternal age, education level, the proportion of primiparity births, and the proportion of multiple births. Previous studies have found that these factors are associated with mortality [18], but we found no indication that the fall in overall mortality is due to changes in the composition of mothers (Table 3). Results for fetal and neonatal mortality give the same conclusion (results not reported). Therefore, it is probable that the driving force behind the development is improvement in maternity care and infant care. This development has been most beneficial for boys.

There is widespread concern that technological developments that contribute to saving more infants have negative side-effects, as they may lead to an increase in the number of new-born infants with health problems [21-23]. Serious health problems at birth reduce the chances of living good and healthy lives without excessive disabilities [24-27]. Since the reduction in mortality has been greatest for boys, we can speculate whether any negative side-effects of the technological development are greater for boys than for girls. This is an issue that deserves attention.

## Conclusions

During the period 1967–2005, the reduction in fetal mortality and neonatal mortality was greatest for boys. The main reason for this is that mortality for boys began at a higher level. The relative reduction in mortality was about the same for both sexes.

## Competing interests

The authors declare that they have no competing interests.

## Authors’ contributions

FC: Conception and design of the study, analysis of the data and writing of the manuscript. JG: Conception and design of the study, acquisition of data and interpretation of analysis. AE: Contributed to conception and design of the study, interpretation of analysis and writing of the manuscript. All three authors read and approved this version of the manuscript.

## Pre-publication history

The pre-publication history for this paper can be accessed here:

http://www.biomedcentral.com/1471-2393/13/101/prepub
